# Provision of antiretroviral therapy for children in Nelson Mandela Bay: Health care professionals’ challenges

**DOI:** 10.4102/phcfm.v10i1.1490

**Published:** 2018-03-12

**Authors:** Margaret Williams, Dalena R.M. van Rooyen, Esmeralda J. Ricks

**Affiliations:** Department of Nursing Science, Nelson Mandela Metropolitan University, South Africa

## Abstract

**Background:**

The human immunodeficiency virus and/or acquired immune deficiency syndrome (HIV/AIDS) pandemic continues to increase in prevalence worldwide, particularly in South Africa, and includes the often overlooked paediatric population. The provision of paediatric antiretroviral treatment (ART) is as essential for children as for adults, and has numerous obstacles, not least of which is lack of decentralisation of facilities to provide essential treatment. Optimising ART, care and support for HIV-positive children, and their caregivers, at public sector primary health care (PHC) clinics is crucial to improve morbidity and mortality rates in children.

**Aim:**

To explore the experiences of health care professionals regarding the provision of ART for children at PHC clinics.

**Setting:**

The study was conducted in six PHC clinics in Nelson Mandela Bay Health District, Eastern Cape, South Africa.

**Methodology:**

The researchers used a qualitative, explorative, descriptive and contextual research design with in-depth interviews. We used non-probability purposive sampling. Data collected were thematically analysed using Creswell’s data analysis spiral. We used Lincoln and Guba’s model to ensure trustworthiness. Ethical standards were applied.

**Results:**

Health care professionals experienced numerous challenges, such as lack of resources, need for training, mentoring and debriefing, all related to providing decentralised ART for HIV-positive children at the PHC level.

**Conclusion:**

Capacitation of the health care system, integration of services, competent management and visionary leadership to invoke a collaborative interdisciplinary team approach is required to ensure that HIV is treated as a chronic disease at the PHC clinic level.

## Introduction

Globally, an estimated 35 million people were living with human immunodeficiency virus (HIV) in 2013; 3.2 million of the 35 million are children under the age of 15 years, over 90% of whom live in sub-Saharan Africa. In the same area, 6000 people were newly infected with HIV and approximately 4200 people died from AIDS, mostly because of inadequate access to antiretroviral (ART) services.^2^ Globally, investments in the HIV and AIDS response have generated positive results. Improved care and treatment options have increased the lifespan of people living with HIV, provided they can access such treatment.^3^ In all low- and middle-income countries, only 23% of children (aged 0–14 years) living with HIV in 2013 received ART, compared to 37% of adults living with HIV. Child mortality statistics indicate that by the end of 2015, almost 6 million children will have died before their fifth birthday – most from preventable causes.^3^

While South Africa faces the world’s largest paediatric HIV epidemic, only one in four children has access to combination ART,^4^ even though South Africa commenced the ART roll-out in 2004.^5^ In fact, South Africa is listed as 58th out of the 100 countries highlighted as having the highest under 5-year-old mortality rates worldwide, and has approximately 360 000 children living with HIV.^6^ Unique considerations exist regarding ART for HIV-infected infants, children and adolescents, a factor that many health care professionals (HCPs) working in HIV settings feel unequipped to confront.^7^

HIV progresses rapidly in infants and young children; therefore, early treatment is vital to their survival. Without treatment, one-third of the HIV-positive children will die in their first year of life, increasing to almost half by the age of 2 years.^2^ Evidence shows that early initiation of antiretroviral drugs in infants and children with HIV can save lives but ART coverage, at 23%, remains too low.^3^ South African paediatric HIV programmes reported persistently high mortality in infants (46.3%) despite large-scale ART roll-out.^4^

Improvement in the early detection of exposed and infected children is of particular importance.^8^ Once children commence treatment, retention on ART and optimal adherence rates are essential to suppress viral replication. The caregivers of HIV-positive children play a crucial role in ensuring that children are compliant with ART medication regimes.^9^ With this in mind, HCPs need to ensure acceptable communication with caregivers in order to ensure that medical treatment for the HIV-positive children in their care is accessed.^10^

The provision of paediatric antiretroviral care, particularly at primary health care (PHC) clinics, has distinct obstacles. One of these is a shortage of staff, which includes staff that are comfortable dealing with children. The researchers noted that in the clinics chosen for the study, only three clinics had a full-time medical practitioner who was employed for PHC, and a sessional (once a week) medical practitioner for ART. The other three clinics depended on a medical practitioner who visited every week (for PHC and chronic illnesses) who neither had the time nor the experience to assist with ART cases – paediatric or adult. None of the medical practitioners was particularly comfortable dealing with paediatric ART cases. Additional challenges include lack of training programmes on the provision of antiretroviral therapy to children, and minimal on-site mentorship of professional staff regarding HIV disease in children. Further challenges are inadequate health care facilities (poor infrastructure) and a lack of resources in general.^11^ The aforementioned results in limitations in confirmatory testing, misclassification of HIV staging in children and lack of initiation of ART.^12^ The lack of capacity in the health care system described previously results in a lack of access to treatment in general, and this is particularly pertinent to HIV-positive children.^11^ The lack of capacity also includes pharmacists who are required to deal with issues related to paediatric ART drug formulations.^4^ In this study, only two clinics had pharmacists, three clinics had pharmacy assistants and one had no pharmacy staff at all.

An additional challenge is the loss to follow up of many HIV-positive infants and children after diagnosis, owing to the centralisation of ART services. There has always been general agreement that children would do better if they could access care closer to home, but lower-level (PHC) facilities have been reluctant to take on paediatric ART initiation and management, leading to malfunction of the decentralisation process in this district.^4,13^ To meet the needs of HIV-positive children in the Eastern Cape, and Nelson Mandela Bay (NMB) in particular, considerable progress is required in optimising decentralisation of ART for children at PHC clinics, preferably using a public health-centred and health care team approach.^4^

## Objective

This study aimed to provide an understanding of challenges for HCPs in providing ART for children attending PHC clinics. This was done by exploring and describing the experiences of HCPs related to their challenges regarding the provision of ART for HIV-positive children at PHC clinics in NMB Health District.

## Research methodology

### Research design

A qualitative, exploratory, descriptive and contextual design was used to understand what supported the phenomenon being studied and share the participants’ experiences.^14^

### Study setting and participants

The researchers purposively included six PHC clinics in NMB in the Eastern Cape from which to interview HCPs. Qualitative research requires in-depth study and smaller samples, chosen from the researcher’s knowledge of the population.^15,16^ The clinics chosen were situated at a considerable distance from the regional hospital previously attended by all children requiring ART in this district. The HIV-positive children specified in this study were pre-schoolers, chosen because the under-five mortality rate in children in South Africa is in the quintile of countries with the highest under-five mortality rates in the world.^3^ The PHC clinics were expressly chosen because the researcher reasoned that the caregivers of HIV-positive children would prefer to utilise the facility that was closest to them to limit the high costs of transport and concurrently time out of work. The researcher interviewed HCPs, namely, 11 professional nurses (N1–N11), 4 doctors (D1–D4) and 4 pharmacists (P1–P4). Professional nurses are responsible for a large portion of the work involved in providing antiretroviral therapy to HIV-positive children, owing to a lack of pharmacists and medical practitioners in PHC clinics.

### Data collection

Nineteen individual in-depth interviews were conducted among the HCPs. Participants were diverse in terms of age, ethnicity, gender, education and occupation (cf. study setting). Fourteen females and five males were interviewed. After ensuring that requisite permission was obtained, the researcher conducted individual interviews with the chosen HCPs. All interviews were conducted and recorded in off-duty times. The central open-ended question to each participant was: ‘How do you experience the provision of comprehensive treatment, care and support as it is provided for HIV-positive children who require antiretroviral therapy at this clinic?’ The researcher sought to obtain an authentic insight into each participant’s experiences which was assisted by using probes. These probes assisted each participant to give more detail in their story telling, and were applied in the form of follow up questions to encourage the participants to expand on their initial answer. Prompting was also used to develop ideas that could be used for elaboration, meaning or reasons.^17^

### Data analysis

Data analysis was conducted simultaneously with data collection according to the steps suggested by Creswell,^16^ in which the analysis process is considered to conform to a general contour, termed the ‘data analysis spiral’. The data spiral consists of four main phases, namely, data management, reading and memoing, describing, classifying and interpreting, and finally visualising and representing the data.^18^ The researchers identified units of meaning which were grouped together to form categories, following which themes were developed.^19^ The data were also analysed independently. The main theme and sub-themes were substantiated by appropriate quotations from the raw data and compared with relevant literature and research.^19^

### Trustworthiness

Credibility, dependability, confirmability and transferability ensure the trustworthiness of qualitative research.^17^ Credibility was ensured by using prolonged and varied field experience, interviewing process, peer review, reflexivity and triangulation.^13^ Transferability is the extent to which the findings are meaningful and was ensured by providing a thick description of the context, participants and findings.^17^ Dependability was ensured by the co-coding procedures, during which two independent coders, plus the researchers, recoded the findings during the data analysis stage.^14^ One of the strategies used to enhance the quality of research is triangulation, which includes the use of various sources to draw conclusions about what constitutes the truth. In this study, triangulation of data-gathering methods and sources were utilised to ensure trustworthiness. In-depth individual interviews were conducted with participants who were aligned within three categories of HCPs, namely, medical doctors, pharmacists and professional nurses. The use of the narratives from miscellaneous participants supported data source triangulation. Furthermore, decisions taken by the researcher were based on the data the field journal produced, and was done to optimise the objectivity or neutrality of the data and ensure confirmability.^17^

### Ethical considerations

Ethical approval was obtained from the Nelson Mandela Metropolitan University’s Research Ethics Committee (Human) prior to conducting the research. The reference number H10HEANUR003 was allocated. The health care professionals who agreed to participate in the study completed an informed consent form. The ethical principles of respect for persons, beneficence and justice were upheld.^1^

## Results

The following main theme and sub-themes, related to the challenges experienced by the HCPs who were interviewed, are presented below:

### Main theme: Health care professionals experienced significant challenges related to providing antiretroviral treatment for HIV-positive children at primary health care clinics

The HCPs highlighted the need for capacitation of the health care system in general and HCPs in particular. They were of the opinion that much needs to be improved at PHC clinic level in order to provide sustainable care and equitable access for all patients, especially children.

### Health care professionals experienced an increased workload at all levels related to the provision of antiretroviral therapy at the clinic level

Most countries that are experiencing high HIV prevalence have weak and poorly resourced health systems. Interventions required to improve health care delivery includes training HCPs, providing incentives to encourage workers to stay, capacitating health care delivery systems and improving infrastructure and institutional capacity.^20^ Participants concur:

‘Well number one they need staff; you need a full complement of staff.’ (D1, medical practitioner, male, 74 years old)

‘I mean there was one pharmacist, one pharmacist assistant and one general assistant doing the work of a pharmacist’s assistant. (P1, pharmacist, female, 64 years old)

There is a drastic shortage of HCPs, especially medical practitioners, and pharmacists in South Africa.^21^ Pharmacists in this study attest to their feelings of being overworked, and thus their inability to provide quality care:

‘Unfortunately, our patient base is too large and we are understaffed in the dispensary so I actually can’t do the counselling [*adherence*] myself.’ (P2, pharmacist, female, 32 years old)

There is equally a shortage of medical practitioners in South Africa, which results in many clinics being run by nurses^21^:

‘Yah there’s no full-time doctor looking after the paediatric cases you know, if they’ve got problems then they always refer them to the adult side.’ (D2, medical practitioner, male, 38 years old)

While the shortage of medical practitioners and pharmacists is highlighted, nursing is also in a desperate situation. Additional to the shortage of nurses is deterioration in quality and productivity^22^:

‘Sisters who were not so committed in the paediatric department often they would just copy the medical practitioners script from previously, not weigh the child, no checking.’ (P1, pharmacist, female, 64 years old)

‘Oh we are short staffed, if people are more understanding you can take the extra load, it’s when people become…when they don’t want to understand and they don’t want to work, and then it becomes a hassle.’ (D1, medical practitioner, male, 74 years old)

Decentralisation of access to health services, a move towards community care and task shifting from medical practitioners to professional nurses and community health workers has not only increased access to ART for patients but has also increased the workload of nurses.^23^

‘The numbers increased and I still did not get any extra help, no other sisters, so from one patient to all these hundreds and thousands I’m still doing the same work, one person.’ (N1, Professsional nurse, female, 37 years old)

A nurse states that she will not assist with paediatric patients requiring ART owing to her already overwhelming workload with the adult ART cases she manages:

‘It’s not gonna really work, it’s not gonna work [*having paediatric patients for ART*], because um too much, the workload is too much.’ (N4, Professional nurse, male 34 years old)

### Health care professionals experienced disharmony within the work environment

While demands for antiretroviral (ARV) therapy expand, the resources to meet these needs are not increasing exponentially, which could lead to disharmony or conflict in the workplace.^24^ Job demands and the lack of job resources can negatively affect work-related well-being.^24^

Increased workload will result from the increased needs of patients, which can lead to stress and conflict and are exacerbated by lack of appreciation and fairness. Concurrent poor relations with co-workers ensue:

‘If people are more understanding you can take the extra load, when they don’t want to work, then it becomes a hassle, we don’t have teamwork.’ (D1, medical practitioner, male, 74 years old)

The HCPs in this study all appeared to feel overwhelmed by their workloads, voicing feelings of being overworked because of the lack of support from colleagues:

‘The other doctor didn’t want to treat the paediatrics and pregnant patients, so a Tuesday is quite busy for me.’ (D4, medical practitioner, female, 37 years old)

‘She’s [*her colleague*] not available, and everybody comes to this side, it’s just not on.’ (N11, Professional nurse, female, 43 years old)

Combined with overwhelming work demands is the lack of job resources with which to complete tasks, which results in workers disregarding work-related norms, and prompting negative behaviour between co-workers^25^:

‘Professional nurses would just copy the medical practitioner’s previous script and copy it WRONG, apart from not weighing the child or adjusting according to weight, but even copy the previous script WRONG.’ (P1, pharmacist, female, 64 years old)

Another major stressor is wasted time and effort,^25^ where HCPs become exasperated at having to double-check the work of others:

‘But the nurses will allow ANYONE, even security [*staff*], to take blood pressures, testing urines and blood sugars, I have to recheck.’ (D1, medical practitioner, male, 74 years old)

A factor that came across succinctly was an apparent lack of understanding among certain professions of the work expected of each grouping. All groups felt overworked:

‘Put more people in the pharmacy, a lot of people are filling posts, getting the money for doing nothing.’ (P3, pharmacist, male, 27 years old)

The criticism (above) may well result in an unproductive atmosphere and job dissatisfaction and ultimately interpersonal conflict:

‘People come to work to earn a salary, not because they want to or enjoy work.’ (D2, medical practitioner, male, 38 years old)

### Health care professionals experienced ineffective management at the clinic level

Health care professionals in this study comment on the clinic management currently in place, but not in a favourable manner:

‘All the service are not done properly, the quality of care is at a very poor level in the clinics.’ (N1, Professional nurse, female, 37 years old)

Many participants are of the opinion that managers at the facility level (PHC clinics) are little more than an addition to the bureaucracy, weak and lacking the motivation to bring about change^26^:

‘At the moment I would almost say what management? There’s no discipline in this clinic.’ (P3 pharmacist, male, 27 years old)

In addition are the frustrations over the apparent inability of managers to deal effectively with clinic-related matters:

‘People are sick, on study leave and there’s nothing catered for that, we barely manage with the staff that we have.’ (N2, Professional nurse, female, 31 years old)

Good managers need to manage the organisational goals, objectives and budgets and achieve harmony among employees^26^:

‘So the clinic is no longer about patient care, its stats, money, political and racial. Patient care – out by the door.’ (N9, Professional nurse, female, 55 years old)

Many of the participants interviewed were angry and frustrated, and if they continued with their current workloads and lack of support they would be open to the repercussions of the unresolved conflict that appeared to be building up. Frustrations were linked to extra duties imposed upon them by others:

‘Every Thursday, paediatric ART clinic, the pharmacist would call in sick, so I was seeing 30 paediatric patients and then I was going to dispense 30 patients’ medicine in the dispensary, it was incredibly frustrating.’ (D4, medical practitioner, female, 36 years old)

‘I just think that if we had dedicated staff there wouldn’t be any of these problems.’ (N9, Professional nurse, female, 55 years old)

‘and you know, there’s a few people that carries 90% of the burden and the rest is just there to, I don’t know how to say it, got a job, because they don’t actually do anything.’ (N6, Professional nurse, male, 34 years old)

### Health care professionals experienced the work environment as being non-conducive to the rendering of adequate care

Challenges are experienced regarding the under-resourced public health sector in which the participants work. Health systems desperately need to be capacitated, particularly owing to the increased demand for health services in the era of HIV/AIDS:

‘We don’t even have oxygen here, then there’s the day that you need the oxygen and it was empty.’ (D1, medical practitioner, male, 74 years old)

Children are not being weighed at every visit because ‘the equipment was faulty’ (N7, Professional nurse, female, 31 years old). This is a concern because the weight ratios/bands in HIV-positive children are the parameter on which the medication dosage is determined:

‘Children get transferred when the weight was this, and they just copy that and they don’t consider what the patient weighs now, then children are overdosed or under dosed.’ (N11, Professional nurse, female, 43 years old)

In order to provide comprehensive PHC of an adequate standard, appropriate infrastructure has to be in place:

‘There just isn’t space, two sisters are working out of one room, infrastructure is quite a big problem.’ (N7 Professional nurse, female, 31 years old)

Post-apartheid South Africa inherited an inequitable health system, which, despite policy changes, has not been reversed. Basic resources are needed to improve the clinic environment:

‘I need people that can get the job done, and computers, printers, a proper log system, you don’t need grand things to make it work, you just need the basics.’ (P4, pharmacist, female, 37 years old)

Health care professionals also have needs that should be met in order to optimise care for patients. One of these is the right to work in a safe environment that is not harmful to health or well-being^27^: ‘Cleanliness [*laughs*], a mop and soap, and space, we need space’ (P4 pharmacist, female, 37 years old), necessity of scaling-up ART, especially for HIV-positive children^21^:

‘[*Children*]..it’s on the cards, but it’s part of the problem, like we don’t have the staff to to to if you see, um there is no way for me to include children in my things, or any other more patients because I am already at my limit….’ (N4, Professional nurse, male, 34 years old)

### Health care professionals experienced incongruence in the interpretation of side effects of antiretroviral medication in HIV-positive children

As a result of ART, HIV has become a chronic, lifelong condition rather than an immediately fatal disease.^28^ However, antiretroviral drugs have documented undesirable effects.^29^ Of concern is that many of the participants attested to a lack of side effects,

‘In my experience the children actually have very few side effects on the drugs.’ (D4, medical practitioner, female, 37 years old)

Which could be for various reasons:

‘Need an integrated system so that can follow up on the history, side effects, currently there’s just no way to do this.’ (P2, pharmacist, female, 33 years old)

HIV-positive children on ART need to be monitored regularly with blood tests for viral load, CD4 counts and thorough clinical assessments. Educational preparation is necessary to ensure that the parent/caregiver is aware of the potential toxicity of the medication and signs related to side effects.^30^ If this is not in place, side effects would not be recorded and followed up:

‘What happens is that unfortunately they only come to the clinic when they are already starting to show the rash, weight loss, and other symptoms (of side effects).’ (P1, Professional nurse, female, 37 years old)

The staff also appear to be relatively uninformed as to the seriousness of reactions to the drugs:

‘I’ve had one patient with um psychosis on Efavirenz and um after we changed from efavirenz to kaletra, she was fine.’ (D3, medical practitioner, female, 33 years old)

‘I had one patient with severe peripheral neuropathy, six years old and weighing 6 kg it was just ridiculous you know.’ (D4, medical practitioner, female, 36 years old)

‘I can say that there haven’t been any major problems um we’ve had one child in fact that we suspect had an abacavir sensitivity and we referred them to hospital, so yah so no major problems with the paediatrics.’ (D4 medical practitioner, female, 36 years old)

According to literature, these are serious side effects. Peripheral neuropathy is a long-term effect of drug therapy.^29^ It should have been noticed at an earlier stage to prevent the negative outcome. Abacavir is associated with a potentially fatal hypersensitivity reaction, which occurs in approximately 5% of HIV-positive children, and is considered a medical emergency. In the aforementioned case, it was treated without any urgency on the part of the medical practitioner.^28^

Another concern over side effects is the seeming confusion between drug reactions and the overlapping effects of HIV. HIV-positive children are at higher risk of neurodevelopment and cognitive impairments^31^:

‘With the ARVs she is not doing well, her work has deteriorated, um I don’t know if it was a previous learning disability.’ (D3, medical practitioner, 33 years old)

‘I’ve seen some other behavioural changes, children are hyperactive all of a sudden, and very inattentive.’ (D3, medical practitioner, 33 years old)

HIV-positive children have considerable risk of delayed mental functioning. The neurodevelopment profile of HIV-positive infants is significantly lower at 12 months of age than that of non-infected children, compromising learning skills.^28^ It is therefore unlikely that HIV-positive children are reacting to the medication in the manner described; damage to the cognitive functioning of the child is probably because of the direct effects of HIV on the neurological system.

Health care professionals need to ensure that they are aware of what constitutes side effects of the medication, and which conditions are to the result of cognitive impairment caused by the disease itself, so that intellectual performance of HIV-positive children can be improved.^21^

‘Professional nurses don’t know what side effects to look out for, they don’t know the opportunistic infections, they can’t prescribe the treatment, they haven’t done antiretrovirals on adults, now we’ve got to ask them to check adherence with the caregiver and the child [*shakes head*].’ (P1, Pharmacist, female, 64 years old)

The lack of ability to ensure optimal treatment, care and support for HIV-positive children in the clinics is reiterated:

‘When it comes to applying the knowledge, I don’t think that’s happening.’ (P4, Pharmacist, female, 37 years old)

Adverse reactions and side effects of antiretroviral drugs occur more frequently than the previous quotations might have indicated. This is verified in the following quotation:

‘The weight loss, the reactions to the treatment, lymph nodes, body aches, gait, the thin limbs and the big tummies we are seeing it.’ (N9, Professional nurse, female, 55 years old)

### Health care professionals experienced apprehension in working with HIV-positive children

Fear of treating HIV-positive children with antiretroviral medication and dealing with the complexities of HIV infection is evidenced in these comments by participants in this study:

‘Um I mean, in this clinic, clinic sisters, and medical practitioners are scared to start therapy with children.’ (P2, pharmacist, female, 33 years old)

Scaling-up ART for HIV-positive children remains impeded, with most provinces struggling to achieve similar targets to those achieved in adults. In the Eastern Cape, HIV-positive children make up less than 10% of all patients on antiretrovirals. Obstacles cited include lack of sufficiently trained staff, laboratory capacity, drug procurement, integration of services and of the knowledge and skills required by HCPs. Health care workers appear to be particularly challenged regarding venesection and children^11^:

‘Shjoe the hardest thing is getting the blood from the babies; oh, it was emotional torture for me.’ (N1, Professional nurse, female, 37 years old)

‘No PCR test was done at six weeks, the reason why she didn’t do it she says she can’t draw bloods on children.’ (N8, Professional nurse, female, 42 years old)

Diagnostic protocols for HIV-positive children are difficult for HCPs because of lack of technical competence and confidence in caring for HIV-positive infants and children. Dedicated training sessions on treating HIV-positive children would assist to overcome the prevalent ‘fear’ of treating HIV-positive children^6^:

‘Preferably have somebody that actually mentor, to get over the fear of paediatrics.’ (D4, medical practitioner, female, 36 years old)

‘if you give the incorrect dose they have far worse side effects, so nurses, unless they are a 100% sure, are very scared to actually prescribe [*for*] paediatrics.’ (P1, Pharmacist, female, 64 years old)

Only 50% of the nurses in PHC clinics had received training in clinical skills, and a mere 38% had formal training in dispensing.^32^ An understanding of the pharmacology of drug interactions in patients on multiple drugs is vital for good clinical practice, and would alleviate fears of working with HIV-positive children on ART:

‘And mostly they don’t know what’s going on, they don’t know how to start children on treatment, they don’t know the medications, the dosages.’ (N8, Professional nurse, female, 42 years old)

‘I’ve seldom come across, except for medical practitioners, other disciplines that’s actually been able to work out doses, they can’t do it.’ (P3, Pharmacist, male, 27 years old)

### Health care professionals experienced concern over the low numbers of HIV-positive children attending the clinic

The clinics where the researcher conducted interviews had minimal HIV-positive children on ART, compared to adults on therapy:

‘I think it’s about 40 children and approximately 1,500 adults.’ (N1, Professional nurse, female, 37 years old)

‘About 1500 adults on ARVs, 100 children.’ (N6, Professional nurse, male, 34 years old)

‘We have got 19 children on register of which we have got five already that are lost to follow-up, adults more than 1500.’ (N7, Professional nurse, female, 31 years old)

‘We have 2400 adults on treatment, currently 17 paediatrics.’ (P4, pharmacist, female, 37 years old)

According to the World Health Organization (WHO) guidelines, HIV-exposed infants should be tested at 6 weeks of age, using RNA polymerase chain reaction (PCR). Staff in resource-constrained settings are assisted by the development of alternative virological tests and dried blood spots on filter paper.^33^ But many clinics are still not testing at 6 weeks of age:

‘So if I’m not here the PCR does not get done, and if there’s no dry blot the PCR does not get done, a patient is 6 months old, what can happen in 6 months? A lot, that is why I am frustrated.’ (N8 Professional nurse, female, 42 years old)

‘It’s a very big concern the child was done PCR at six weeks and was positive, no ARVs was given but the child is on Bactrim, they are not following the guidelines.’ (P2, pharmacist, female, 33 years old)

‘I mean the two weeks that the municipal clinic was on strike was an eye opener for us, because we got so many patients, PCRs are not done, Nevirapine is sometimes not given, prophylaxis is not given.’ (N7 Professional nurse, female, 42 years old)

There have been considerable developments in scaling-up HIV/AIDS therapy, and some integration between areas of the health sector, such as maternal, new-born and child health; tuberculosis; and PHC.^28^ Regrettably, ART access for HIV-positive children does not improve, which concerns participants:

‘You know I thought, I, I thought it would be more by now, you know? You listen to the newspaper and the TV and they say so many households are child run, parents have died, where are the children?’ (D1, medical practitioner, male 74 years old)

‘Let me say on the em, hindsight, because what I’ve noticed is that we don’t have that many children on the ARV programme.’ (D3, medical practitioner, female, 33 years old)

‘Where are the kids? We haven’t got any children on antiretrovirals.’ (N5, professional nurse, female, 55 years old)

From the figures in the previous quotations, and evidence from reports, fewer than 25% of HIV-positive children are currently accessing ART. Intensified efforts and strategies are required to increase interventions for HIV-positive children, in quantity and quality. HIV response in resource-constrained areas should be evidence informed, so that interventions are linked to local epidemiology to ensure maximum impact^28^:

‘The one doctor just refused and said he’s not going to see the children, so it was all on the other doctor; I just think I could have refused many things in my life but you do it for the patients’ sake, because they need to benefit they are the sick ones.’ (N1, professional nurse, female, 37 years old)

According to Eley,^34^ ‘The care of children younger than 6 months with advanced HIV infection is one of the most technically demanding clinical challenges that I face’. Early initiation of ART, improved interventions for opportunistic diseases and appropriate access to intensive care is required, but staff with limited support structures are ‘scared’ of treating HIV-positive children:

‘Ja and I’ve seen now when the other doctor tried to write up a prescription he did it incorrectly, so I can understand why he’s scared, I would be scared.’ (N6, professional nurse, male, 34 years old)

‘At many clinics, they are refusing to see paediatrics because they don’t have the staff.’ (D1, medical practitioner, male, 74 years old)

The apparent lack of knowledge and confidence among health care workers to manage HIV-positive children causes reluctance to test and treat young patients^31^:

‘The medical practitioners when they are not familiar with an area they don’t wanna do it. They want to do something that will take quickly; a child is going to take time.’ (N9, Professional nurse, female, 55 years old)

‘That’s the hard part, it’s not nice emotionally to see the children, cos sometime you can see they not well, and there’s nothing else you can do.’ (N2, Professional nurse, female, 31 years old)

A few participants considered what would enhance the provision of ART at the clinics. They thought that the clinic environment should be more child friendly, and that staff working with HIV-positive children should enjoy working with children, in order to ensure that mother and child could relax and benefit from therapy. This is in keeping with many studies that suggest child friendly environments^31^:

‘Proper counselling areas, play areas, let them get familiar with the medication, just make it more child friendly.’ (N9 Professional nurse, female, 55 years old)

‘I enjoy that, seeing the whole family.’ (N1 Professional nurse, female, 37 years old)

### Health care professionals experienced lack of adherence to antiretroviral medication

The challenge with ART is to ensure that caregivers understand the importance of total adherence to medication in order to suppress a virus that has proven resilient to control. Virological failure increases sharply for patients with adherence rates lower than 90%^31^:

‘Just the problem is the defaulters and the default rate now, it is sky-high.’ (D3, medical practitioner, female, 33 years old)

‘So they will come back late, adherence is very important, you don’t miss ARVs for two days or three days, that’s what’s happening all the time and we still don’t know.’ (D2, medical practitioner, female, 36 years old)

‘When you default your chances of building up um resistance increases and your fatality increases, people are ignorant about the whole HIV issue.’ (P2, pharmacist, female, 33 years old)

Adherence to antiretroviral medication is uniquely challenging in HIV-positive children; organisational skills are required to ensure daily child dosing, particularly when the child is uncooperative. HCPs need to be aware of the psychological and logistical complexities in the administration of medication to HIV-positive children to be able to assist the parents/caregivers.^35^ It also seems to be the norm to place this task onto lay HCPs, in the guise of task shifting.^36^

While this is understandable, the training and supervision of this task should be outlined and formalised so that parents/caregivers are informed of their responsibilities towards the HIV-positive children in their care. Participants in this study relate to the challenges in adherence in HIV-positive children:

‘Patients who are HIV-positive will come in for their own medication but they don’t care about the children’s medication, if it’s time to wait for a child script they will leave it and come back and fetch it in a day or three, no interest you know.’ (P2 pharmacist, female, 33 years old)

‘I’ve got actually two children failing on treatment, both of them are not adherent, there are issues there.’ (N2 Professional nurse, female, 31 years old)

Reasons for missing medication doses in HIV-positive children that were presented in this study included forgetting, child refusing or vomiting (no re-dosing); running out of medication (through lack of finance); distance; long waiting times at the clinics; incorrect dosing by the caregiver; confusion between carers where there is more than one; caregiver’s attitude towards ART; and disclosure, which elicits support, or lack of it, when the caregiver fears stigmatisation:

‘With the children they are being looked after by other family members so there our adherence is a problem because if that guardian is working they send somebody else or they don’t come for the treatment.’ (N8 Professional nurse, female, 42 years old)

‘Caregivers give the incorrect dosage, I will ask; “How much are you giving?” they will say; “Haai man sister, I don’t know”; then you find they are giving 8 instead of 6 mls.’ (N10, Professional nurse, female, 30 years old)

‘We have placed our lay health counsellors within those services [*ART for children*], so they at least counsel, but we need to understand that lay health counsellors are very limited in training so the actual professional nurse has to almost verify that information, almost have to make sure you’ve been given the right information, time it’s the time factor.’ (D4, medical practitioner, female, 36 years old)

## Discussion

HIV infection in HIV-positive children is a life-threatening disease. Without treatment, and specifically adherence to treatment, HIV-positive children in resource-poor settings have a 45% – 59% mortality rate by 2 years of age.^35^ ART for HIV-positive children is an evolving discipline, which should be specialised owing to the intricacies in working comprehensively with children of all ages who require treatment, care and support for one of the most complex diseases of our time. Up until now, the focus has been on the provision of medications and management of physical disease. The rapid progression of paediatric HIV disease has highlighted the need to expand treatment, for this cadre of patients, to date with limited success. Certainly, better performance is required from health care systems in general, especially in the Eastern Cape, South Africa.

According to WHO,^6^ health systems need to perform better in order to respond to current challenges and the expectations of communities for better performance. Primary health systems need to ensure social relevance with leadership that is participatory.^36^ Leadership and management positions should only be given to those who are competent and are not afraid to be held accountable. Only after these changes are implemented can the real work begin and the current downward cycle in the health care services be reversed.^6^

Health care institutions should practise crisis-probing into the operational and management structures to ascertain weaknesses and flaws.^37^ Poorly managed workplace conflicts can wreak havoc on work productivity, demoralise staff, increase turnover and damage relationships.^38^ Health managers are the key to conquering health delivery constraints but clearly establishing the capacity and effectiveness of health management is not straightforward.^38^

If employees are unhappy, their work suffers. Managers of health care clinics need to improve their knowledge and skills in terms of organisational details, particularly linked to the highly educated workforce they are dealing with, wherein interrelationships can become complex. Competent managers are needed so as to ensure interprofessional collaboration to guarantee optimal treatment for all patients.^26,33^

The antiretroviral programme, essentially doctor driven, is undergoing changes to mandate and expand the role of nurses so that they can legitimately initiate treatment and prescribe antiretrovirals. However, this will increase the nurses’ workload.^26,33^ South Africa currently has the largest antiretroviral roll-out programme in sub-Saharan Africa. It warrants mentioning that the enormity of providing ART to more than 1 million people would challenge any health care system.^39^

Strategies are required to improve adherence to ART by providing requisite care and support, especially with regard to patient information specific to the needs of children with HIV. The challenges at PHC level incorporate a need for staff to update their knowledge in order to manage children with HIV effectively.^39^

Comprehensive paediatric care must be offered to drastically reduce the health inequities for children at PHC clinics. This includes ensuring that psychosocial factors, as they are related to the child, caregiver and family functionality, and the resources available to the family are explored, highlighted and solutions sought. However, given the considerable challenges related to shortages of HCPs, social workers, infrastructure and miscellaneous resources, support and care is often overlooked, especially for children, despite being as important as the drugs, or treatment, aspect.^40,27^

Undeniably an acceleration of paediatric Nurse Initiated Management of Antiretroviral medication (NIMART) is imperative. Clinical mentoring and further training in clinical skills and pharmacology would benefit all practitioners.^26,41^ The participants in this study verified that they require training, mentoring and support in working with infants and HIV-positive children requiring ART.

Undoubtedly, teamwork is required to ensure that each member of the interdisciplinary teams working in PHC recognises the values, expertise, perspectives and experience of one another. Gauging the comments of the various HCPs in this article, there is little collaboration, mutual understanding or teamwork occurring in the PHC clinics in NMB.^42^

## Recommendations

It is recommended that all interdisciplinary team members receive updates in knowledge and skills in order to effectively optimise comprehensive treatment, care and support for HIV-positive children requiring ART at PHC clinics.^43^ Particular emphasis should be placed upon committing to quality health care outcomes for all patients.

Strengthening the roles and responsibilities of clinic managers should be ensured. Managers are clearly under pressure to adequately perform management roles and should be enabled to develop leadership skills in order to adequately manage and maximise the use of all resources, including staff members.^35^ Guaranteeing the provision of support services such as home-based and community-based health services, and their links with fixed PHC facilities in particular, is critical to good health outcomes, particularly related to adherence to medication.^43^

Case management is suggested as a solution to the low coverage of treatment for children with HIV because it encompasses assessment, planning, implementation, monitoring and review and, when properly implemented, can strengthen outcomes for caregivers and children, through integrated and coordinated service delivery.^44^ Case management encompasses family-centred care because the process includes identifying the family’s needs and ensuring that requisite services are coordinated, and managed in a systematic way, to the benefit of the child and the family.

The interdisciplinary team members should apply self-reflective practices. Reflection is a part of a cycle of learning for each person where one generally looks back on an experience and reviews what has been learned in order to identify areas for practice improvement.^45^ In addition, establishing a climate of awareness and knowledge is recommended so that all HCPs are mindful of the need to engage in the latest evidence-based guidance to clinical practice in order to provide the best care for patients and their families.^46^

## Conclusion

HIV disease in children is a chronic, manageable condition in all countries with access to ART. It is time to clarify treatment for HIV-positive children and assist HCPs in South Africa to become knowledgeable and confident in treating younger patients. Judging from the responses of the participants in this study, the workforce at the PHC level in NMB needs to be strengthened to provide sustainable care and equitable access, a task requiring competent management with leadership skills.

Capacitation of the health care systems was highlighted by HCPs who indicated excessive workloads, with too few staff and too many patients to provide the endorsed patient with the family-centred approach required for the optimal treatment of HIV-positive children. Health care professionals discussed their frustrations with poor work ethic and lack of commitment by many staff members towards patients. Scathing opinions were presented by many of the HCPs underpinning their discontent at the ineptitude of management in particular and the health care system in general. Specifically, management in most clinics was deemed non-existent or weak, lacking motivation or ability. There were few participants who felt that their manager could control the functioning of the clinic in order to achieve work satisfaction for the staff, and optimal efficiency of the ART service, specifically for children.

Health care professionals indicated that guidelines are generally poorly adhered to, medication is not always given accurately and adherence levels are notably poor in HIV-positive children. Participants suggested the need for strategies with which to adequately motivate caregivers to ensure and maintain adherence to ARTs on behalf of the children in their care. Overall, there is insufficient care for children with HIV at PHC clinics related to the ubiquitous lack of resources in all facets of the health care services.
